# 

**Published:** 1868-06-01

**Authors:** 


### CONTENTS:

FOREIGN CORRESPONDENCE:

Letter from Paris. By W.......... 347

CORRESPONDENCE:

Letter from Philadelphia. By E. K. H.......... 351

Observations in the Treatment of Sterility. By H. Webster Jones, M.D.......... 352

Extra-Uterine Fœtation. By L. T. Strother, M.D.......... 355

EDITORIAL.......... 360

BOOK NOTICES:

Researches in Obstetrics. By J. Matthews Duncan.......... 361

Felix Von Niemeyer's Clinical Lectures on Pulmonary Phthisis.... 361

The Indigestions; or, Diseases of the Digestive Organs Functionally Treated. By Thomas King Chambers.......... 361

On Diseases of the Skin. By Erasmus Wilson, F. R. S.......... 361

Rush Medical College.......... 362

